# Reporting of cumulative antimicrobial susceptibility testing data, including antibiograms

**DOI:** 10.4102/sajid.v40i1.775

**Published:** 2025-09-29

**Authors:** Colleen Bamford

**Affiliations:** 1 Division of Medical Microbiology, Faculty of Health Sciences, University of Cape Town, Cape Town, South Africa

## Introduction

Knowledge of local cumulative antimicrobial susceptibility test (AST) data is often used to guide empiric treatment of patients with suspected infections, particularly those with severe infections or septic shock, before or in the absence of individual microbiology results. Local treatment protocols are typically based, at least in part, on such knowledge, while expansion of antimicrobial stewardship programmes has led to an increased awareness of the need for these data.

The Southern African Journal of Infectious Diseases (SAJID) frequently receives submissions containing cumulative AST data from various settings. Although publication of these articles may be useful, authors must clarify the aim and purpose of their manuscript, including asking how their local data differ from other settings and how it adds value to readers elsewhere.

Most importantly, such articles must be based on accurate data and appropriate methodology. The Clinical and Laboratory Standards Institute (CLSI) is a not-for-profit organisation that develops consensus-based international laboratory standards and testing guidance. The CLSI guideline for the presentation of cumulative AST data (CLSI guideline M39) is widely used and quoted, with its most recent update in 2022.^1^ Unfortunately, many CLSI guidelines are available by subscription only, although a recent freely available article highlighted the newer areas in the latest CLSI guideline M39.^2^ We present a simplified outline of the fundamental aspects of the CLSI guidelines on how to present cumulative AST data for contributors to the SAJID and other relevant journals. These recommendations were reviewed and endorsed by the Federation of Infectious Disease Societies (FIDSSA) prior to publication.

We also recommend the inclusion of a clinical microbiologist familiar with the study setting in the study team from the early stages of study development to ensure that data are appropriately analysed.

Note that the primary aim addressed in the CLSI guideline and in this article is the presentation of cumulative (AST) data to guide selecting empiric therapy. Alternative analyses may be more appropriate where cumulative AST data are used for other purposes, such as identifying emerging resistance or monitoring resistance trends in public health initiatives. These alternatives are addressed briefly in the recent CLSI update.^1^

## Definitions

The latest CLSI guideline provides updated definitions to clarify terminology.^1^ ‘Cumulative AST data reports’ refer to all reports based on analysis of compiled AST results for a defined period, showing the susceptibility as a percentage of a single species or organism group (e.g. Enterobacterales) to each antimicrobial. ‘Antibiogram’ refers to such results generated from a single health care facility according to CLSI methods. An ‘enhanced antibiogram’ stratifies the susceptibility data by specific parameters, e.g. by specimen source (blood or urine, etc.) or by patient location (ICU versus ward, etc.). The term ‘cumulative antibiogram’ is no longer recommended. ‘Multifacility antibiogram’ refers to isolates from multiple facilities where additional concerns about differences in testing methods may apply.

## Valid laboratory results

One should include only final verified laboratory results. Unusual results should be checked – there may be undetected errors in data entry or reporting. Occasionally, misleading organism–antimicrobial combinations, such as organisms with intrinsic resistance to an antimicrobial, are included erroneously in the reports. For example, enterococci are intrinsically resistant to cephalosporins. Another example is when antimicrobial combinations with *in vitro* laboratory susceptibility do not translate into clinical efficacy, e.g. aminoglycosides and early-generation cephalosporins are not recommended for Salmonella infections. Both the CLSI^3^ and the European Committee on Antimicrobial Sensitivity Testing (EUCAST)^4,5^ provide information on intrinsic resistance and recommended antimicrobial agents.

Ideally, testing should be performed in an ISO15189-accredited laboratory using validated methods aligned with laboratory testing guidelines. It is essential that laboratories use the correct contemporary interpretive breakpoints. In South Africa, most microbiology laboratories, including all public sector laboratories, use CLSI breakpoints.

## Data extraction

A key principle is that only data from clinical samples should be included. Samples collected for surveillance purposes from patients without clinical suspicion of infection, e.g. routine surveillance respiratory or urine samples collected from ICU patients, should be excluded. Only pathogens should be included, while commensal and/or contaminant bacteria should be excluded. Classification of bacteria as pathogens or commensals depends on specimen type and clinical condition. Details of common commensals are found in the updated version of the National Healthcare Safety Network (NHSN) commensal list.^6^

Furthermore, if multiple isolates of the same organism are isolated from a patient during the study period, only the first isolate should be included (even if the isolates come from different specimen types). This reduces the bias that would be introduced from multiple isolates derived from sicker or longer term patients. If the study period is longer than 1 year, one isolate per year can be included.

In the South African public sector, data from National Health Laboratory System (NHLS) laboratories are usually extracted from the laboratory information system (LIS) by a central data-handling unit. These data have the advantage of including test results generated by both automated and manual testing systems and may include limited metadata, such as age, gender and specimen type, but without clinical information. There may also be other limitations; for example, depending on the LIS minimum inhibitory concentration (MIC), data may not be extractable, limiting further analysis. The use of both automated and manual testing methods for the same organism does introduce potential variability.

## Data analysis

We recommend reporting the percentage of susceptible isolates (as opposed to the percentage resistant). Under CLSI testing, isolates reported as ‘intermediate’ are not grouped with the susceptible category. However, this may differ for laboratories using (EUCAST) methods, which have different definitions and different breakpoints. The authors should state which AST methods and reporting systems were used.

Clinical and Laboratory Standards Institute guidelines recommend that a minimum of 30 isolates per organism and/or organism group be reported together with 95% confidence intervals or credible intervals to ensure statistical validity. Small sample sizes result in too much potential variability. A recent study calculated the potential level of error in antibiograms by selecting samples of different sizes from a larger pool of susceptibility tests and suggested that a minimum of above 60 isolates is required to give an error rate of less than 5% if the resistance rate for the organism-antimicrobial combination is between 40% and 60%.^7^ Small sample size is a major and frequent problem in generating local antibiograms. Failing to recognise this error frequently leads to over-interpretation of insignificant changes in percentage susceptibility. Potential solutions include combining similar organisms, e.g. all *Klebsiella* species or all Enterobacterales, or extending either the period of analysis or catchment area.

It is essential that all organisms are tested against a full panel of antimicrobials. The practice of selective testing, whereby only organisms resistant to initially tested antimicrobials are tested against broader spectrum or reserve antimicrobials, generates unreliable results. Therefore, state the number deemed susceptible (*n*), the total number of isolates tested (*N*) and the percentage susceptible (*n/N*) for each organism–antimicrobial combination. As a rule of thumb, do not report any antimicrobial tested against less than 90% of isolates. Alternatively, if authors wish to include selective reporting data, these should be reported separately and clearly defined as such with limitations acknowledged. For laboratories practising cascade reporting to clinicians (i.e. reporting only narrow-spectrum, first-line antimicrobials for susceptible organisms to promote antimicrobial stewardship), the cumulative data analysis must be based on the full panel of antimicrobials tested.

## Limitations

Antibiograms based on routine laboratory surveillance have inherent limitations for consideration before interpretations and conclusions are drawn from the data.

Antibiograms are dependent on the submission of samples (presumably well collected) as requested by clinicians whose test request practices may vary by person and place. Antibiograms derived from LISs neither provide correlation with clinical outcomes nor differentiate community from hospital-acquired infections. Unless restricted to sterile sites, e.g. from blood or cerebrospinal fluid, there is no differentiation between infection and colonisation.

Antibiograms are only part of the decision-making process for empiric therapy. Other important considerations include the severity and site of infection as well as the likelihood of resistant organisms in a patient based on factors such as the duration of hospitalisation, previous infections or colonisation and previous antimicrobial therapy.

In addition, statistically significant changes may not necessarily translate into meaningful clinical significance and changes in empiric choices. The latest CLSI guideline does briefly address establishing threshold levels or resistance rates at which empiric therapy recommendations should change. However, thresholds probably vary according to the severity of infection, the risk of spread to other people if inadequately treated and the availability of alternatives.

## Recommendations for publication

In addition to the general guideline on the generation of cumulative AST data reports, the following recommendations are essential for articles based on data submitted to SAJID for peer review:

- Briefly describe the setting of the study to contextualise the results.
- Include details of the laboratory AST testing methods as well as methods for data extraction and analysis. This helps to ensure reproducibility of studies and enables comparisons between different studies. For studies analysing AST changes over time or differences between testing laboratories, any changes or differences in laboratory methods or interpretive breakpoints must be highlighted.
- Describe the selection criteria used, including any deduplication approach, and, if necessary, explain why this approach was appropriate for the research question.
- Analyse results as fully as possible, e.g. reporting not only categorical percentage susceptibility but also available MIC data, including percentiles, interquartile range, median or Mininimum Inhibitory Concentration for 50% of organisms (MIC_50_) as well as MIC_90_.
- Include appropriate tests for statistical significance in the analysis.
- Highlight the overall findings in relation to previous relevant studies. Mention any limitations and biases.

## Conclusion

Generation and dissemination of cumulative AST data reports can provide useful information supporting antimicrobial stewardship. The Southern African Journal of Infectious Diseases encourages researchers to analyse such data and, where appropriate, to submit for publication. We hope that these guidelines, summarised in Box 1,^1^ will facilitate high-quality and accurate reports.

BOX 1 A summary of Clinical and Laboratory Standards Institute recommendations on analysis and presentation of cumulative antimicrobial susceptibility test data.

**Table:** 

Summary of recommendations
**Only include:** Clinical or diagnostic isolates Final verified test results The first isolate of a species from an individual patient during the analysis period, regardless of source or susceptibility profile Species with AST data for a minimum of 30 isolates Antimicrobial agents tested routinely against all isolates. Calculate % susceptible from results for all isolates, irrespective of whether they were reported or suppressed from patient reports and include 95% confidence intervals
**For manuscripts, also include:** Full details of methods used for AST, data extraction and analysis Full analysis of data, including appropriate tests for statistical significance Discussion of limitations and biases

*Source*: CLSI. Analysis and presentation of cumulative antimicrobial susceptibility test data. CLSI guideline M39. 5th ed. Clinical and Laboratory Standards Institute; 2022.

AST, antimicrobial susceptibility test.
